# 

**Published:** 1846-10

**Authors:** 


### BOOKS RECEIVED FOR REVIEW.

1. The Sanative Influence of Climate. By Sir James Clark, Bart. Fourth edition. London, 1846. 8vo, pp. 412. 10s. 6d.

2. Practical Surgery. By Robert Liston. Fourth edition, London, 1846. 8vo, pp. 582. 22s.

3. Observations on the Principle of Vital Affinity, as illustrated by Recent Discoveries in Chemistry. By W. P. Alison, M.D., F.R.S.E. (From the Transactions of the Royal Society of Edinburgh.) Edinburgh, 1846. 8vo, pp. 23.

4. The Open Surgery Question considered. By W. J. Preston. London, 1846. 8vo, pp. 25.

5. Meade's Manual for Students preparing for Examination at Apothecaries' Hall. Second edition. London, 1846. 8vo, pp. 586. 10s. 6d.

6. The Economy of the Animal Kingdom, considered Anatomically, Physically, and Physiologically. By Emanuel Swedenborg. Translated from the Latin by the Rev. A. Clesseld, M.D. Two vols. 8vo, pp. 574-426.

7. Verbrennung und Athmen, chemische Thätigkeit und organisches Leben. Von Fr. Nasse. Bonn, 1846. 8vo, pp. 154.

8. Introductory Lecture to a Course of Military Surgery. By Sir George Ballingall, M.D. Edinburgh, 1846. 8vo, pp. 23.

9. Fragments of Medical Science and Art. An Address. By H. J. Bigelow, M.D. Boston, 1846. 8vo, pp. 54.

10. A Series of Essays on Inflammation and its Varieties. Essay I. The Natural History of the Disease. By Henry Clutterbuck, M.D. London, 1846. 8vo, pp. 67.

11. Three Reports of the Joint Deputation of the Society of Apothecaries and the National Association. London, 1846. 8vo, pp. 46. 1s.

12. Analysis of the Evidence in Favour of the Constant Supply System. By J. Wicksteed, Esq. London, 1846. 8vo, pp. 46.

13. Inaugural Dissertation on Yellow Fever. By Hugh Bone, M.D. Edin. 1846. 8vo, pp. 85.

14. Historical and Critical Remarks on the Operation for the Cure of Cataract. By A. Watson, M.D. Edinburgh, 1845. 8vo, pp. 35.

15. On the Treatment of Strictures of the Urethra. By James Briggs, Surgeon. London, 1845. 8vo, pp. 62. 2s. 6d.

16. On the Health of Towns, as influenced by Defective Cleansing and Drainage. By W. A. Guy, M.B. London, 1846. 8vo, pp. 48. 1s.

17. On the Incubation of Insanity. By Forbes Winslow, M.D. London, 1846. 8vo, pp. 23.

18. On the Injurious Effects of Mineral Poisons in the Practice of Medicine. By H. Prater, M.D. London, 1846. 8vo, pp. 96.

19. Lectures on the Urine, and on the Pathology, Diagnosis, and Treatment of Urinary Diseases. By J. Aldridge, M.D. Dublin, 1846. 8vo, pp. 80. 2s. 6d.

20. Kissingen. By N. A. Travis, M.D. Francfort. 12mo, pp. 32. 1s.

21. Homœopathy, Allopathy, and Young Physic. By John Forbes, M.D. Philadelphia, 1846. 8vo, pp. 120.

22. The Use of the Body in relation to the Mind. By G. Moore, M.D. London, 1846. 8vo, pp. 431. 9s.

23. The Surgical, Mechanical, and Medical Treatment of the Teeth. By J. Robinson. London, 1846. 8vo, pp. 320. 10s.

24. Medical Report of the House of Recovery and Fever Hospital, Boyle street, Dublin. By G. A. Kennedy, M.D. Dublin, 1846. 8vo, pp. 52.

25. Experimental Researches on the Food of Animals, with Remarks on the Food of Man. By R. D. Thomson, M.D. London, 1846. 8vo, pp. 195.

26. Elements of Physics. By C. F. Peschel. Translated from the German by E. West. 3 vols. London, 1846. 8vo.

27. Practical Observations on Mineral Waters and Baths. By E. Lee. London, 1846. 8vo, pp. 134. 4s. 6d.

28. Dr. Hooper's Physician's Vade Mecum. By Dr. Guy. New edition. 1846. 8vo, pp. 524. 10s. 6d.

29. A Medical Topography of Tunbridge Wells. By R. H. Powell, M.D. London, 1846. 8vo, pp. 174. 3s. 6d.

30. Clinical Collections and Observations in Surgery. By W. P. Ormerod, Surgeon. Lond. 1846. 8vo, pp. 312.

31. Fever Physiologically considered. By D. M. Reed. London, 1846. 8vo, pp. 262. 10s.

32. The Antidotal Treatment of Cholera. By R. Parkin, M.D. London, 1846. 8vo, pp. 48. 2s. 6d.

33. The Microscopical Anatomy of the Human Body. By A. H. Hassall, F.L.S. 8vo. 1846. Parts I and II. 2s. 6d. each.

34. A Practical Treatise on the Diseases of Children. By J. M. Coley, M.D. London, 1846. 8vo, pp. 467. 14s.

35. Notes on the Epidemic Cholera of India. By A. H. Kennedy. London, 1846. 8vo. 10s. 6d.

36. Handbuch der Pathologie und Therapie. Von Dr. C. A. Wunderlich, Professor zu Tübingen. Stuttgart, 1846.

37. The Harveian Oration, delivered before the Royal College of Physicians. By John Elliotson, M.D., F.R.S. London, 1846. 8vo, pp. 70, 2s.

38. The Blood, Anatomically, Physiologically, and Pathologically considered. The Warneford Prize Essay for 1845. By C. E. Joseph, M.R.C.S. London, 1846. 8vo, pp. 126.

39. Observations on the Edinburgh Pharmacopœia. By Richard Phillips, F.R.S., &c. Lond. 1846. 8vo, pp. 56.

40. The Mineral Waters of Kreutznach. By J. E. P. Prieger, M.D. Translated by O. Prieger, M.D. London, 1846. 8vo, pp. 92. 3s. 6d.

### INDEX TO VOL. XXII

OF THE

### BRITISH AND FOREIGN MEDICAL REVIEW.

PAGE

Alison, Dr. S. on alterations of the heart . . . . . . 234
Amputation of limbs, statistics of . 52
Amputation at the hip-joint, treatise on . . . . . . . 111
Anatomy, pathological, Dr. Vogel's 323
Antagonism of phthisis and fever 213
Aneurism, Mr. Liston's treatment of 373
Aphorisms in medicine, Dr. Pauli's 53
Apoplexy, its connexion with heart-disease . . 408-20
influence of age on . 421
treatment of . . . 428
Dr. Huss's report on . 461
Arachnitis, history and treatment of 296
Army, on the field-carriage of . 225
Arteries, Mr. Quain's great work on 556
Arteries, Professor Porta's work on 89
experiments on the ligature of . . . 91
Porta, on the torsion of . 102
Arteries and nerves, plates of . 231
Association, Provincial, transactions of . . . . . . 319
Asthma, grinders', Dr. Favell on . 320
Atlas of plates, Dr. Mouatt's . 227
Auscultation and percussion, Gunzburg on . . . . . . 232
Austria, history of foundlings in . 338

Balfour, Dr. his report on homœopathy . . . . . . 567

Bartlett, Dr. on the progress and prospects of medicine . . 235

Baynard, Dr. his anticipation of Liebig . . . . . . 433

Bladder, cancer of . . . 305

prolapsed, case of . . 320

Blood and nerves, Dr. Heidler on . 219

Blood and urine, analysis of . 223

Blood, pathological relations of . 326

PAGE

Blood-vessels, Mr. Liston on injuries of . . . . . . 372
Bones, hereditary brittleness of . 49
Mr. Liston on the injuries of 369
Bonnet, M. on diseases of the joints . . . . . . 62
Books received for review . 292-594
Brain, on the physiology of . . 488
its special functions . . 516
Boudin, Mr. on the causes of phthisis and fever . . . . . 211
Bowditch, Dr. his young stethoscopist . . . . . . 209
Brain, inflammation of the membranes of . . . 385
on the physiology of . 488
comparative anatomy of . 494
and heart, relation of the diseases of . . . 408
Breast, Sir B. Brodie on tumours of 167
on the diseases of . . 156
female, Mr. Tuson on . 173
Brodie, Sir B. his lectures on surgery 160
Bronchocele, operation for . . 50
Burrows, Dr. on disorders of the cerebral circulation . . . 408

Cancer, M. Lebert on . . . 32
Dr. Walshe's treatise on . 293
Carbonic acid, on the quantity exhaled . . . . . . 189
Carotids, effects of ligature of . 417
Carpenter, Dr. on the structure of the brain . . . . . 501
Cerebellum, its functions . . 529
comparative anatomy of 535
Cerebral circulation, Dr. Burrows on 408
Cerebrum, not the seat of sensation 504
its functions . . 511
Cerebro-spinal affections and heart-disease . . . . . . 425

PAGE

Cholera, its treatment by homœopathy . . . . . . 580

Children, on the diseases of . . 548

China, Dr. Wilson's medical notes on 179

Circulation, cerebral, Dr. Burrows on 408

Clark, Sir James, his influence of climate . . . . . . 228

Climate, Sir James Clark on the influence of . . . . . 228

Cold-water cure, treatises on . 428

Coley, Dr. on diseases of children . 548

Colic, its treatment by homœopathy 580

Collections, medical, Mr. Maclure's 224

College of physicians cannot make *Doctors* . . . . . . 549

Coma, causes of . . . . 418

Combe, Dr. A. on phrenology . 230

Corrrespondence with the editor on "Young Physic" . . 243

Cox, Mr. on amputation at the hip-joint . . . . . . 111

Crosse, Mr. his case of prolapsed bladder . . . . . . 320

Currie, Dr. on cold affusion . . 434

Diarrhœa, its treatment by homœopathy . . . . . . 579

*Doctor of Medicine*, on the title of 549

Dropsy, pathological anatomy of . 325

Drowning, Sir B. Brodie on . . 164

Dysentery and fever in China . 185

its treatment by homœopathy . . . 579

Encephalon, cancer of . . 309
Escharotics, Mr. Liston on the use of 368
Esdaile, Dr. on mesmerism in India 475
Evans, Dr. his lectures on phthisis 85

Farcy, Mr. Percival on . . . 47

Fergusson. Dr. his notes and recollections . . . . . . 551

Fever, its treatment by cold water 446

and dysentery in China . 182

history and mystery of its treatment . . . 552

gastric, its treatment by homœopathy . . . 576

intermittent, its treatment by homœopathy . . ib.

Fleischmann, his homœopathic treatment . . . . . 567

Floyer, Sir John, his work on cold water . . . . . . 432

Foundlings, history of, in Austria . 338

Fractures, ununited, Sir B. Brodie on 166

Gastrodynia and gastritis, diagnosis of 297
Genital organs, cancer of . . 306

PAGE

Geological relations of phthisis and fever . . . . . 211

Gall, his doctrine of the cerebellum 534

Ganglia, cephalic, their functions . 495

Germany, state of homœopathy in 565

Gilchrist, Dr. on the natural treatment of wounds . . . 238

Gintrac, M. intra-thoracic tumours 216

Glanders and farcy, Percival on . 35

Glanders, symptoms of . . 37

varieties of . . . 39

causes of . . . 40

pathology of . . 45

Glasgow, the vital statistics of . 215

Grinders' asthma, Dr. Favell on . 320

Gums, cancer of . . . . 294

Günzburg on percussion . . 232

Hannover, Dr. on the exhalation of carbonic acid . . . 189
Hassall, Mr. his microscopic anatomy 552
Headache in diseases of the heart 425
Heart and brain, on the relation of diseases of . . . . . 408
Heidler, Dr. on blood and nerves . 219
Hemorrhagia cerebri, diagnosis of 387
Hernia, and the operation for . 51
Mr. Teale's treatise on . 193
on dividing the stricture in 377
Hip-joint, treatise on the amputation at . . . . . . 111
Homœopathic treatment, editor's remarks on . . . . . 592
Homœopathy, its history in Germany. . 565, 567
report on . . 567
Horaczek, Dr. on icterus . . 311
Hospital, homœopathic, at Vienna 570
Huss, Dr. his report on diseases . 459
Hydrocephalus acutus, case of . 403
Hydropathy, treatises on . . 428
its physiological and therapeutic effects . . . 441

Icterus, or bilious dyscrasy, treatise on . . . . . . 311
Indians, their treatment by water 435
Inflammation, Dr. Lebert on . 19
of the membranes of the brain, . . 385
Injections, use of in hydarthrosis, &c. 71
Insanity, Dr. Jacobi on the form of 1
cases of by Dr. Jacobi 3, 5, 7, 9
Dr. Jacobi on the phenomena of . . 12, 16
on the treatment of . 55
use of narcotics in . 55
Dr. Steward's classification of . . . 59

| | PAGE |
|---|---|
| Insanity, moral treatment of | 60 |
| Dr. Thurnam's statistics of | 349 |
| relative liability to | 360 |
| Intestines, cancer of | 299 |
| Irritability, nervous, from heart disease | 425 |
| Jacobi, Dr. on the forms of insanity | 1 |
| account of his writings | 2 |
| Joints, treatise on the diseases of | 62 |
| causes of diseases of | 63 |
| treatment of diseases of | 65 |
| Mr. Liston on the diseases of | 370 |
| Kidney, cancer of | 303 |
| Lanzani, his work on cold water | 431 |
| Lebert, Dr. his pathological physiology | 18 |
| Leeson, Mr. on the heart and lungs, &c. | 221 |
| Liebig travestied | 221 |
| Limb, on the shortening and lengthening of in hip-cases | 78 |
| Lindley, Mr. his vegetable kingdom | 554 |
| Lips, cancer of | 293 |
| Liston, Mr. his practical surgery | 366 |
| Lithotrity, Mr. Liston on | 376 |
| Liver, cancer of | 300 |
| Login, Dr. on army-field-carriage | 225 |
| Lungs, cancer of | 301 |
| Maclure, Mr. his collectanea medica | 226 |
| Mammæ, on the diseases of | 156 |
| hypertrophy of | ib. |
| Mania without illusion, case of | 3, 7 |
| with delirium, case of | 5, 9 |
| Medicine, its progress and prospects | 235 |
| Melzer, Dr. his history of foundlings | 338 |
| Mericourt, M. on diseases of the breast | 156 |
| Mesmerism in India | 475 |
| Microscopic anatomy, Hassall on | 552 |
| Milk abscess | 157 |
| Morbid growths, Mr. Liston on exploration of | 374 |
| Mouatt, Dr. his Hindustani anatomy | 227 |
| Mühry, Dr. on homœopathy in Germany | 565 |
| Mütter, Dr. his edition of Liston's lectures | 379 |
| Narcotics, their use in insanity | 55 |
| Nature, on excessive trust in | 557 |
| Neisser on inflammation of the membranes of the brain | 355 |
| Nerves and blood, Dr. Heidler on | 219 |
| arteries, plates of | 231 |
| Newport, Mr. on the structure of the brain | 499 |
| Noble, Mr. on the physiology of the brain | 488 |
| Nose, Dr. Mütter's formation of a | 381 |
| Œsophagus, cancer of | 295 |
| Operations, employment of mesmerism | 475 |
| Osteoma frontalis, case of | 462 |
| Ovaries, cancer of | 307 |
| Paget, Mr his report on anatomy and physiology | 261 |
| Pathology, brute, its importance | 35 |
| Pauli, Dr. his observations in surgery | 49 |
| Percival, Mr. on glanders and farcy | 35 |
| Peschel, M. his elements of physics | 553 |
| Phillips, Mr. his treatise on serofula | 124 |
| Phrenology, its nature and uses | 230 |
| criticism on its doctrine | 488-517 |
| Physics, Peschel's elements of | 553 |
| Physiology of the brain, Mr. Noble on | 488 |
| Phthisis, Dr. Evans's lectures on | 85 |
| and scrofula, are they identical | 132 |
| Physician, on the deserts and duties of | 115 |
| his *saper-fare* or *savoir-faire* | 117 |
| Physiology, pathological, Dr. Lebert on | 18 |
| Pneumonia, Dr. Huss on | 468 |
| its treatment by homœopathy | 581 |
| view of cases of | ib. |
| Poisons, mineral, Dr. Prater on | 545 |
| Polycholia | 312 |
| Porta, Professor, on ligature of arteries | 89 |
| Practitioners, portraits of various leading | 121 |
| Prater, Dr. on mineral poisons | 545 |
| Predisposition to scrofula, on the | 144 |
| Pressure in the treatment of aneurism | 377 |
| vascular, within the cranium | 413 |
| Priessnitz, his system of medicine | 436 |
| Products, morbid, pathology of | 329 |
| Pus, Dr. Lebert on | 21 |
| pathology of | 330 |
| Quain, his great work on the arteries | 556 |
| Ratier, M. on syphilis | 226 |
| Rees, Dr. on blood and urine | 223 |
| Report on anatomy and physiology, by Mr. Paget | 261 |
| Retreat, the York, history of | 363 |
| Retzius, Professor, on the structure of the brain | 502 |

PAGE

Rheumatism, its treatment by homœopathy . . . . . 577

Sava, Dr. on the duties of the physician 115
Scarlatina, its treatment by homœopathy . . . . . . 581
Skoda, Professor, his treatment of pneumonia . . . . . 593
Scrofula, Mr. Phillips's treatise on 124
statistics of . . . 125
signs of . . . 127
and phthisis, are they identical 132
prevalence of, in different countries . . . 138
on the causes of . . 145
treatment of . . . 150
Scrofulous matter, its characters and source . . . . . 130
Spinal irritation, Dr. Huss on . 403
treatment of . 466
Stethoscopist, the young . . 209
Steward, Dr. on insanity . . 55
Stockholm, report of diseases in . 459
Stomach, cancer of . . . 296
Strangulation, Sir B. Brodie on the effects of . . . . . . 161
Surgery, Dr. Pauli's researches on . 49
Sir B. Brodie's lectures on 160
practical, Mr. Liston's . 366
Mr Liston's lectures on . 379
application of mesmerism in 475
Symonds, Dr. on excessive trust in nature . . . . . . 557
Syphilis, Sir B. Brodie, on mercury in 169
M. Ratier on . . . 226

Taylor, Mr. on the temperature of the earth . . . . . . 229
Teale, Mr. his treatise on hernia . 193

PAGE

Temperature of the earth and sea . 229
Thurnam, Dr. his statistics of insanity 349
Tongue, cancer of . . . . 294
Torsion of arteries, Professor Porta on 101
Transactions of provincial association 319
Tubercle, anatomy and chemistry of 27
Tumours, pathological physiology of 29
intra-thoracic, treatise on 216
pathology and classification of . . . 334
Tuson, Mr. on the female breast . 173
Typhus fever, Dr. Huss on . . 269
its treatment by homœopathy . . . 573

Urine, Dr. Rees on the analysis of 223
Urology, Dr. Leroy-d'Etiolles on . 233
Uterus, polypus and inversion of . 51

Vegetable kingdom, Professor Lindley's 554
Vis medicatrix naturæ, requires guiding . . . . . . 557
Vienna, homœopathic hospital of . 567
Vital statistics of Glasgow . . 215
Vogel, Professor, his pathological anatomy . . . . . . 323

Water-cure, treatises on . . 428
Watt, Dr. on the statistics of Glasgow . . . . . . 213
Walshe, Dr. on cancer . . 293
Williams, Dr. on insanity . . 55
Wilson, Dr. his medical notes on China . . . . . . 179
Wounds, on the natural treatment of . . . . . . 238

"Young Physic," correspondence respecting . . . . 240

END OF VOL. XXII.

C. AND J. ADLARD, PRINTERS, BARTHOLOMEW CLOSE.

